# Reprogrammable Permanent Shape Memory Materials Based on Reversibly Crosslinked Epoxy/PCL Blends

**DOI:** 10.3390/molecules25071568

**Published:** 2020-03-29

**Authors:** Iker Razquin, Alvaro Iregui, Lidia Orduna, Loli Martin, Alba González, Lourdes Irusta

**Affiliations:** 1 POLYMAT, Department of Polymer Science and Technology, University of the Basque Country UPV-EHU, PO Box 1072, 20080 Donostia/San Sebastian, Gipuzkoa, Spain; irazkin14@gmail.com (I.R.); alvaroiregui92@gmail.com (A.I.); lidia.orduna@ehu.eus (L.O.); alba.gonzalez@ehu.es (A.G.); 2 Macrobehaviour-Mesostructure-Nanotechnology SGIker Service, Faculty of Engineering, University of the Basque Country UPV-EHU, Plaza Europa 1, 20018 Donostia/San Sebastian, Gipuzkoa, Spain; loli.martin@ehu.es

**Keywords:** epoxy, polycaprolactone, shape memory, reprogrammable permanent shape

## Abstract

Epoxy/Polycaprolactone (PCL) blends cured with a conventional diamine (4,4′-diaminodiphenylmethane, DDM) and with different amounts of a disulfide containing diamine (4, 4´-dithioaniline, DSS) were prepared through melting. The curing process was studied by FTIR and differential scanning calorimetry (DSC) and the mechanical behavior of the networks was studied by DMA. The shape memory properties and the recyclability of the materials were also analyzed. All blends showed a very high curing degree and temperature activated shape memory effect, related to the glass transition of the epoxy resin. The PCL plasticized the mixture, allowing tailoring of the epoxy glass transition. In addition, in the blends cured with DSS, as a consequence of the disulfide exchange reaction, the permanent shape could be erased and a new shape could be reprogrammed. Using this strategy, reprogrammable permanent shape memory materials were obtained.

## 1. Introduction

Shape memory materials are those that can store a temporal shape. This shape is maintained until an appropriate stimulus is applied and the material recovers its original permanent shape [1,2]. For example, shape memory metallic alloys [3] present excellent shape memory properties but have a low deformation ability and must be processed at high pressure and temperature. The polymer materials, in part, overcome these drawbacks. 

There are many polymer materials that present temperature-activated shape memory properties [4]. These materials can store a temporal shape and when a switching temperature is surpassed, they can recover their original shape. Generally these materials are constituted by two components—one of which is responsible for maintaining the integrity of the material and the other is a switching component that causes a change in the material stiffness when the switching temperature is surpassed [5]. The stable component can be either a thermoplastic or a thermostable material. The switching component is a material that presents a thermal transition such as a glass transition or a melting point. Generally speaking the thermostable materials present better chemical, thermal, and mechanical properties [6]. In addition, the properties of these materials can be adjusted by varying the crosslinking density and the curing conditions.

Epoxy resins have been widely employed as shape memory thermostable materials [7,8,9,10]. The low molecular weight epoxy prepolymer is cured to give rise to a crosslinked rigid structure and to obtain shape memory properties, another component that must be incorporated in order to give the material its switching abilities. This component can be introduced in the chemical structure of the curing agent or as a modified epoxy resin [11,12]. Some examples of shape memory epoxy resins containing poly-ε-caprolactone (PCL) can be found in literature where some authors have chemically linked both components [13]. In these works, the melting of the PCL acts as a switching transition so that the maintenance of the crystalline structure of the PCL plays an important role in the shape memory process [14,15,16,17]. Other authors have used epoxy/PCL physical blends [18,19]. 

However, epoxy resin-based shape memory materials have some flaws from the environmental point of view. The difficulty of being reprocessed, motivated by their thermostable nature is perhaps the most relevant negative aspect and constitutes an obstacle in the attempt to involve these resins in the paradigm of the "Circular Economy" [20]. In an attempt to overcome this obstacle, some authors have incorporated reversible curing agents to crosslinked resins to give rise to recyclable networks. These materials are referred to as “reversible adaptable networks” (CAN) and can be classified as dissociative or associate linkage (vitrimers) [21,22,23]. Thus, Diels Alder type moieties [24,25] have been introduced in epoxy resins to give rise to dissociative CANs that show healing and recycling abilities [26,27]. Anhydride cured epoxy resins have also been studied as associative vitrimeric polymer networks [28].

Disulfide exchange reaction [29] has also been explored to obtain shape memory polyurethanes [30], crosslinked poly-ε-caprolactone [31] and epoxy resins. For example, disulfide linkages have been introduced in epoxy prepolymers [32] or via crosslinking using 4.4’-dithiodianiline [33]. Hu and co-workers [34] have also used this curing agent to crosslink a bio-based epoxy resin. This last material shows shape memory properties but in addition, due to the disulfide linkages, the permanent shape of this material can be changed, which can be of great interest in the development of novel smart materials. Recently, these reversible curing agents have been used in epoxy resin/Diels Alder containing polyurethane blends [35]. The permanent shape of these materials can also be changed.

It is clear that the introduction of reversible linkages into epoxy-based shape memory materials not only allows the recycling of the resin but also promotes the obtaining of materials with permanent shape-changing abilities. However, in the reported works the monomers must be chemically modified. Taking this into account, in the present work we have obtained shape memory materials using commercially available bisphenol A diglycidyl ether (DGEBA), which was blended with a high molecular weight polycaprolactone. The mixture was cured using disulfide containing curing agents and blends with conventional curing agents, to obtain cost-effective shape memory materials. Thanks to the disulfide moieties, the material showed the ability to permanently change the shape.

## 2. Results and Discussion

Different samples were prepared by melt mixing of an epoxy resin with high molecular weight PCL and the curing agents by using the conditions described in the experimental part. Before curing, the samples containing DDM had pale-yellow, transparent, and homogeneous characteristics that were maintained after curing. Samples cured with DSS showed similar characteristics but a deeper yellow color.

### 2.1. Characterization of Samples with DDM

**Figure 1:**
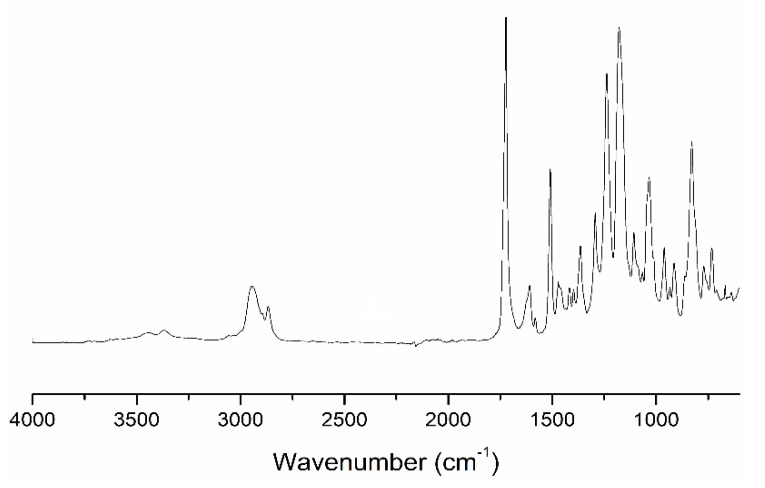
Infrared spectrum of the sample 5050DDM before curing.

The curing process was studied using Fourier transform infrared (FTIR) spectroscopy. Figure 1 shows the infrared spectrum of the sample 5050DDM, before curing.

In the spectrum, the characteristic bands of both DGEBA and PCL could be found. Thus, the main absorptions were the 3390 cm^−1^ and 3211 cm^−1^ stretching vibrations of the amine corresponding to DDM; 1732 cm^−1^ stretching vibration of the C=O corresponding to PCL; 1615, 1511, and 1429 cm^−1^ stretching vibration of the aromatic ring; 1249 and 1115 cm^−1^ stretching vibration corresponding to the DGEBA ether bond; 833 cm^−1^ out of plane bending of the aromatic ring of DGEBA; and 914 cm^−1^ out of plane bending of the epoxy group.

**Figure 2:**
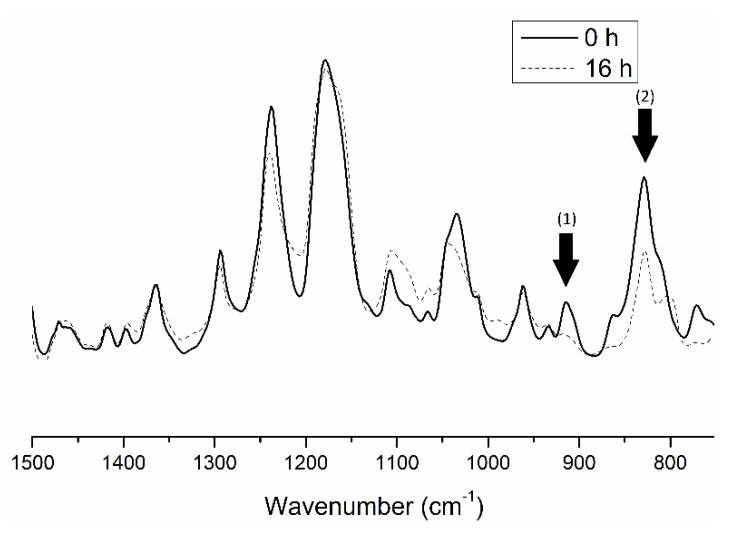
Infrared spectrum of the sample 5050DDM before and after curing for 16 h at 100 °C.

In order to follow the kinetics of the epoxy curing, the film was cut into small pieces that were placed in an oven at 100 °C. Samples were removed at different reaction times and the infrared spectra were recorded. Figure 2 shows the scale-expanded infrared spectra of the sample 5050DDM, before and after curing in the oven for 16 h. The infrared spectra at different reaction times for this composition could be found in the Supplementary Material (Figures S1 and S2).

As observed in Figure 2, the absorbance of the band at 914 cm^−1^ (1) (related to the epoxy group) was considerably reduced after being placed in the oven. An example of the calculation method used to determine the absorbance of this band is shown in Figure S3. It was considered that the area of the band was not affected by the surrounding bands and therefore no curve resolution program was used to determine the area of the band. The absorbance of this band normalized with the absorbance at 833 cm^−1^ (2), which was used to calculate the conversion (Equation (1)) [36].

(1) $$X=\left(1-\left(\frac{{\left(\frac{A_{915}}{A_{830}}\right)}_{t}}{{\left(\frac{A_{915}}{A_{830}}\right)}_{t_{0}}}\right)\right)\times 100$$

where A_915_ t and A_830_ t are the areas of the bands of the epoxy ring and the out of plane bending of the aromatic ring (which does not change during the curing process) at a time t, and A_915_ t_0_ and A_830_ t_0_ are the initial areas of these bands.

**Figure 3:**
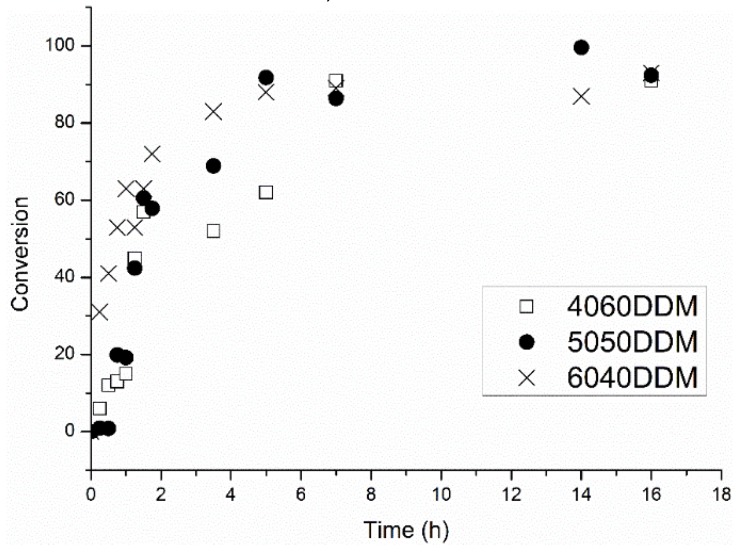
Conversion vs. time calculated from the FTIR spectra for samples containing different bisphenol A diglycidyl ether (DGEBA)/ Polycaprolactone (PCL) ratios cured with DDM.

Using Equation (1), the conversion was calculated for different reaction times. Figure 3 shows the results of this calculation for samples with different composition, DGEBA/PCL cured with the DDM curing agent (the data can be found in Table S1).

As observed in Figure 3, in all samples, the conversion increased with time reaching a final value close to 90%. In addition, the initial slope of the curves was higher as the DGEBA content increased. This fact was attributed to the steric hindrance imposed by the PCL. According to literature, in this system hydrogen bonding interaction between the carbonyl of the PCL and the hydroxyl group of the DGEBA can take place, increasing the system compatibility. Therefore, the dilution of the miscible PCL in the epoxy resin could be responsible for the observed behavior [37].

**Figure 4:**
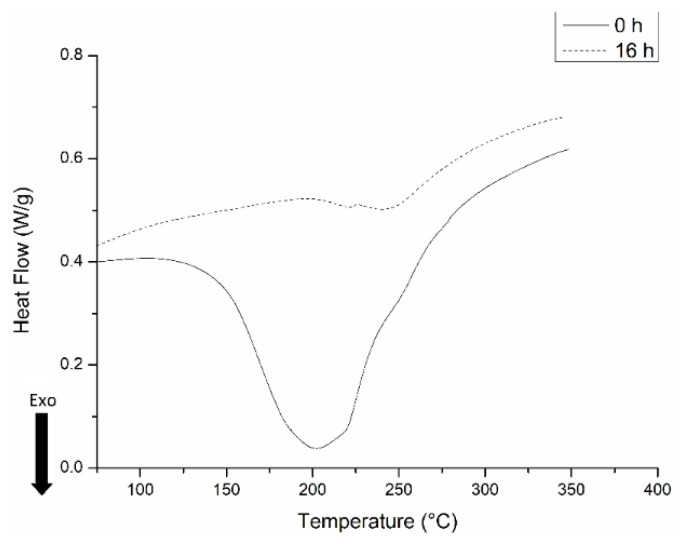
Differential scanning calorimetry (DSC) thermogram of sample 5050DDM, before and after 16 curing hours.

The curing process was followed by differential scanning calorimetry (DSC), to corroborate the FTIR results. Figure 4 shows the DSC thermogram of the sample 5050DDM, before and after curing at 100 °C, for 16 h.

As observed, the non-cured sample showed a curing exotherm at 200 °C. However, as a consequence of the high conversion of the crosslinking reaction, the curing exotherm was clearly reduced in the sample cured in the oven.

The conversion of the curing reaction (X) was calculated by DSC. The curing enthalpy measured at each reaction time was related to the initial curing enthalpy, according to Equation (2).

(2) $$X=1-\frac{\Delta H_{t}}{\Delta H_{t_{0}}}\times 100$$

where ∆$H_{t}$ is the enthalpy of the sample at different curing times and $\Delta H_{t_{0}}$ is the enthalpy of the non-cured sample. The values of the enthalpy and conversion with time for all samples can be found in the Supplementary Material (Table S2).

The DSC data (Figure S4 Supplementary Material) corroborated those obtained by FTIR. Thus, all samples gave rise to a curing degree close to 90% and the curing rate increased with the epoxy content.

**Figure 5:**
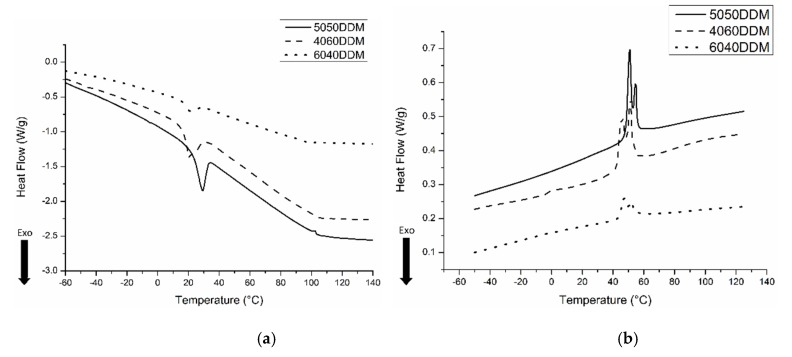
(**a**) DSC cooling scans for different composition of DGEBA/PCL blends, cured with DDM for 16 h; and (**b**) subsequent heating scans after the cooling run shown in (**a**).

The crystallinity of the PCL in different composition DGEBA/PCL blends was studied by DSC. Figure 5 shows the cooling and subsequent heating scans of the samples containing different DGEBA/PCL ratios cured with DDM. As observed in the heating scan, all samples showed two peaks close to 50 °C that were related to the PCL melting. The presence of the two peaks was attributed to the interference of the crystallization imposed by the epoxy, which gave rise to a fraction of less perfect PCL crystals that melted at lower temperatures [38]. In addition, the crystallization temperature of the sample 5050DDM was higher than that of the other samples. Higher size of the PCL domains can be in the origin of these behavior. The reason of this is not clear but it is possibly related to the phase diagram of the epoxy/PCL blend.

The crystallinity of the samples was calculated using the enthalpy obtained in the DSC heating run and by taking the enthalpy for pure 100% crystalline PCL (135.37 J/g) into account [39]. The results are shown in Table 1.

As observed, compared to pure PCL, the blends showed very low crystallinity values. However, the samples containing more PCL showed a slightly higher value. This result evidenced that there was a high interaction between the epoxy and PCL that hindered the crystallinity of the latter, which was in accordance with literature data where this blend is described as miscible [37].

**Figure 6:**
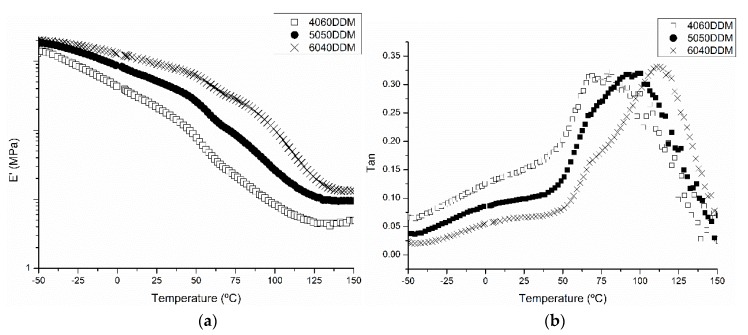
Modulus (**a**) and Tan δ (**b**) vs. temperature for different DGEBA/PCL composition samples cured with DDM.

To determine the modulus and the glass transition of the cured samples, dynamical mechanical thermal analysis (DMA) were performed for the different DGEBA/PCL composition samples cured for 16 h, at 100 °C, with DDM. The results are shown in Figure 6.

As observed, the elastic modulus (Figure 6a) decreased with temperature, showing two slope changes related to the PCL melting at around 60 °C and at around 100 °C related to the glass transition of the material. At higher temperatures the modulus was stabilized, which was a consequence of the crosslinked nature of the material.

The tan δ values (Figure 6b) showed that the maximum was shifted to higher temperatures with a decrease in the PCL amount. Pure epoxy resin cured with DDM showed a Tan δ value of 180 °C, see Supplementary Material (Figure S5). The compositions, which had less PCL, had higher values of the glass transition. This result could be explained by taking into account that the PCL can plasticize the epoxy network giving a homogeneous mixture and reducing the epoxy glass transition. Therefore, the glass transition of the epoxy could be tailored with the PCL concentration, which is of great interest in developing shape memory materials.

### 2.2. Characterization of Samples Cured with DDM/DSS Mixtures

**Figure 7:**
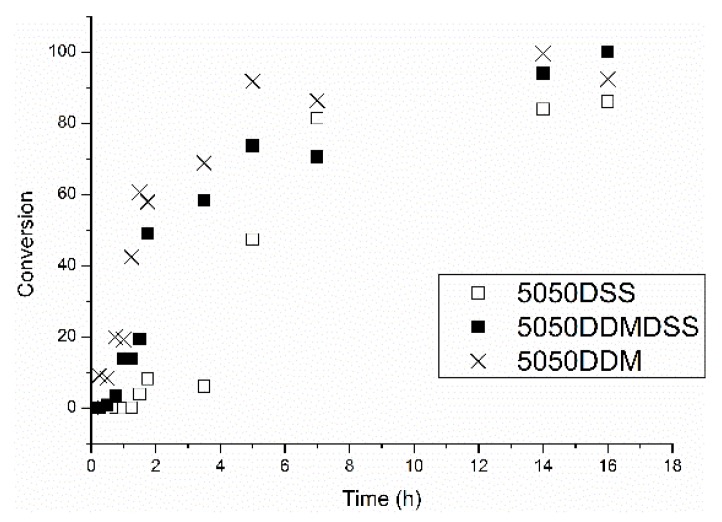
Conversion vs. time calculated from FTIR spectra for 50/50 DGEBA/PCL blends cured with different DDM/DSS ratios.

Taking these results into account, we decided to select the 50/50 DGEBA/PCL blend in order to perform the experiments with the disulfide-linkage-containing curing agent. Figure 7 shows the evolution of the conversion during the reaction time studied by FTIR for the 50/50 DGEBA/PCL blends cured with different composition of DDM/DSS as curing agents. The infrared absorbance and conversion for all samples can be found in Table S3.

As observed in Figure 7, in all samples, the conversion increased with time, reaching a final conversion close to 90%. In addition, the initial slope of the curves was higher as the DDM content was increased.

The curing process was also followed by DSC and the results are shown in the Supplementary Material (Table S4, Figure S6).

The DSC data corroborated those obtained by FTIR. Thus, all samples gave rise to a curing degree close to 90% and the curing rate increased with the DDM content. In addition, the DSC thermograms showed that in samples containing DSS, no melting endotherm was obtained. The result implies that in this sample PCL was unable to crystallize. It is clear that in our system, the epoxy resin is an obstacle for the PCL crystallization. The crystallization of the PCL is possible in PCL domains with enough size, which only happens in a phase-separated mixture. In fact, if this system is cured with another curing agent, the blend is phase-separated and the PCL shows a high crystallinity degree [37]. Therefore, the absence of any melting endotherm in the samples cured with DSS can be indicative of a higher phase mixing of this system (compared to the samples cured with DDM).

**Figure 8:**
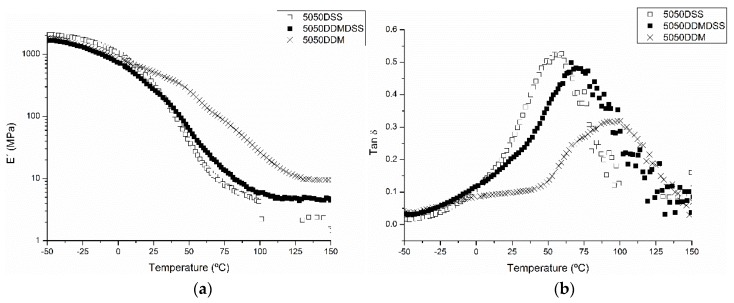
Modulus (**a**) and Tan δ (**b**) vs. temperature for sample 50/50DGEBA/PCL cured with different DDM/DSS ratios.

To determine the modulus and the glass transition of the cured samples, dynamical mechanical thermal analysis (DMA) were performed for the different 50/50 DGEBA/PCL blends cured with different DDM/DSS ratios. The results are shown in Figure 8.

As observed in Figure 8a, the modulus decreased with the addition of DSS. This result matches with literature data, where the lower modulus was also attributed to the presence of labile disulfide bonds [34].

The tan δ values are shown in Figure 8b. The compositions that had a higher tan δ value in the maximum was the 5050DSS. This result was related to the presence of the dynamic disulfide bonds that gave mobility to the network, increasing this value. In addition, the maximum of the tan δ value shifted to higher temperatures when increasing the DDM content. This result can be explained by taking into account that Tan δ value of the pure DGEBA sample cured with DDM is close to 200 °C, while the value of DGEBA cured with DSS is 150 °C (Figures S5 and S7).

### 2.3. Morphology of the Material 

**Scheme 1:**
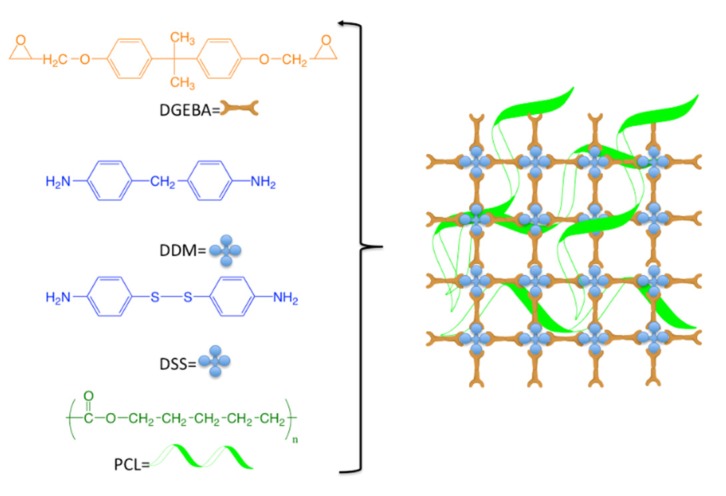
Scheme of the structure of the material.

In the final material, the cured epoxy resin formed a network and the PCL was linked by hydrogen bonding interactions between the carbonyl of the PCL and the epoxy hydroxyl. Scheme 1 shows a schematic representation of the structure of the material.

**Figure 9:**
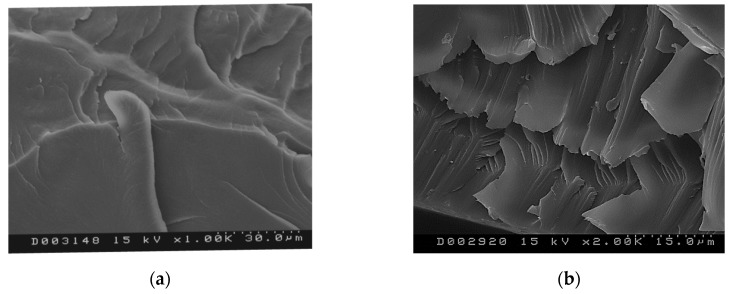
SEM image of the sample 5050DDM. (**a**) ×1.00 k magnification and (**b**) ×2.00 k magnification).

The morphology of the material was studied by SEM. In Figure 9, the morphology of the system 5050DDM is shown.

The images showed a homogenous morphology, which is in accordance with previous publications about PCL/epoxy mixtures cured with DDM [37]. The morphology was very similar for the different DGEBA/PCL compositions cured with DDM and with different mixtures of DDM/DSS (see Supplementary Material Figure S8), which evidenced that all samples showed a homogeneous morphology.

### 2.4. Shape Memory Properties

**Figure 10:**
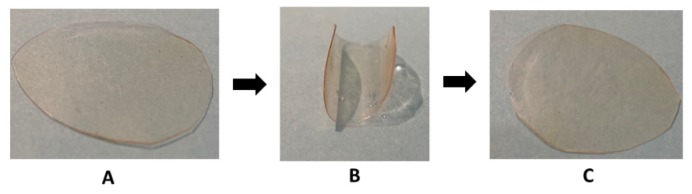
Shape memory cycle for the sample 5050DDM. (**A**): Permanent shape. (**B**): Programmed shape. (**C**): Recovered shape.

The shape memory properties were studied by visual testing. In order to select the switching temperature of the experiment and, as the samples presented very low or no crystallinity, the glass transition value of the samples was considered. However, the glass transition of all samples was not the same and it increased with the DDM content. As the aim of this paper was to study the behavior of the disulfide containing samples, the glass transition of this sample (5050DSS) was considered to select the switching temperature, which was set at 80 °C. Figure 10 illustrates the shape memory cycle for the sample 5050DDM.

As observed, a temporal shape could be programmed when heating at 80 °C and cooling at room temperature, (Figure 10B). However, when heating at 80 °C, the original shape was recovered (Figure 10C), showing that this sample presented shape memory properties. This behavior was observed in all prepared samples regardless of the PCL amount and the chemical nature of the crosslinking agent. It was interesting highlight that in the literature for most of the PCL blends that present shape memory properties, this behavior is related to the PCL melting/crystallization process. However, in the present work the crystallinity degree of the PCL was very low in DDM-cured samples and was negligible in samples cured with DSS. According to this, the shape memory of these systems was related to the epoxy glass transition. The introduction of the PCL plasticized the system, reducing the glass transition, and making the material less stiff, allowing a temporal shape to be programmed.

In addition, some of our samples contained a curing agent with dynamic covalent bonds (DSS). As observed in the DMA results, this network had a higher mobility because of the dynamic nature of the crosslinker. In order to change the permanent shape of the material, it was given a new shape by placing it in the oven at 100 °C, for 5 h. The idea was that the internal stress was relaxed by disulfide exchange at higher temperatures and a new permanent shape could be programmed for a pure epoxy material, as demonstrated in the literature [34].

**Figure 11:**
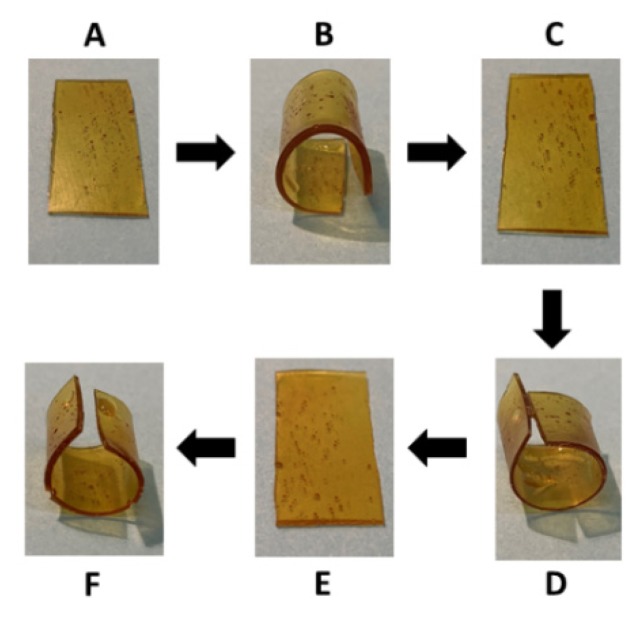
Reprogrammable shape memory cycle for sample 5050DSS. (**A**): Initial permanent shape. (**B**): First temporal shape. (**C**): Recovered shape. (**D**): Reprogrammed permanent shape. (**E**): Second temporal shape. (**F**): Second recovered shape.

In order to demonstrate this ability, a new temperature cycle was applied to these samples (Figure 11).

As shown in Figure 11, starting from an initial permanent shape (A), a new temporal shape at 80 °C (B) that was fixed at room temperature, was programmed. Then, when heating at 80 °C, the initial permanent shape was recovered (C). When a new shape was given to the sample and the sample was placed in an oven at 100 °C for 5 h, a new permanent shape could be programmed (D). Next, a new temporal shape (E) could be programmed by heating at 80 °C and then cooling. The second permanent shape was recovered when heating at 80 °C (F). This result demonstrated that when heating in the oven at 100 °C for 5 h or more, a new permanent shape could be programmed to the material. Using this strategy, we observed that the materials 5050DDMDSS and 5050DSS were able to set a new permanent shape. However, it is important to highlight that for the 5050DDM composition it was not possible to reprogram the permanent shape because of the more permanent nature of the obtained network.

**Figure 12:**
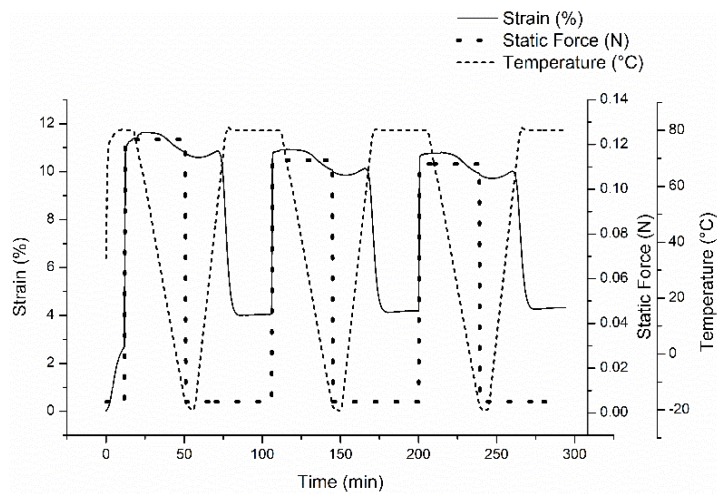
Dynamical mechanical thermal analysis (DMA) shape memory test for sample 5050DSS.

The shape memory effect was also analysed by DMA in tensile mode for three consecutive cycles. The results for the sample 5050DSS are shown in Figure 12.

As observed, in all cycles, the sample was able to fix a strain when lowering the temperature and recovering the shape when heating.

DMA test was performed for all formulations and from these data, the fixity and recovery ratios were calculated using Equations (3) and (4), described in the experimental part. The results of all samples can be found in the Supplementary Material (Figures S9 and S10). The results are summarized in Table 2.

In the first cycle, all samples showed high fixity ratios. On the contrary, in the first recovery cycle, the samples did not return to the programmed strain. However, in the following cycles, the programmed strain was recovered. Some authors have pointed out that after the first straining process, entanglement decoupling could result in more homogeneous properties, after the first shape memory cycle [40]. The recovery ratio in the first cycle and the fixity ratios were lower in the samples cured with DDM. This result was not easy to explain. The lower glass transition of the sample cured with DSS could be in the origin of this effect. It must be remembered that, as the aim of this paper was to study the shape memory properties of the samples containing disulfide bonds, the shape memory experiment was optimized by taking the glass transition of sample 5050DSS into consideration. Therefore, at the employed switching temperature sample 5050DDM, was probably below its Tg and sample 5050DDMDSS, was very close to its Tg. In the transition regime between the glassy and rubbery states, when a stress was applied, the strain crept continuously with time and when the stress was removed, a part of the strain recovered, affecting the fixity ratio. In addition, as the sample was between the glassy and rubbery state, the recovery process was also affected [41].

### 2.5. Recycling Properties

**Figure 13:**
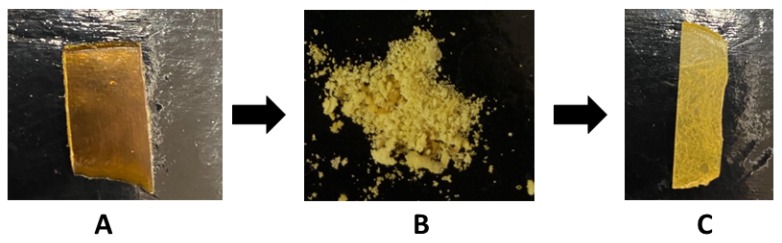
Reprocess cycle of the material 5050DSS. (**A**): Pristine sample. (**B**): Grinded sample. (**C**): Recycled sample.

The possibility of recycling the samples containing the disulfide curing agent was checked, as illustrated in Figure 13.

After the film was ground, it was placed in a mold and a pressure of 150 bars was applied for 1 h at 125 °C. Figure 13C shows the image of the obtained film. As observed, the ground material was able to produce a film, which confirmed that the material could be reprocessed due to the disulfide linkage [33,34]. This was exclusively possible in the sample that only contained DSS curing agent. The other samples cured with DDM or with DDM/DSS mixtures were not able to produce a film.

**Figure 14:**
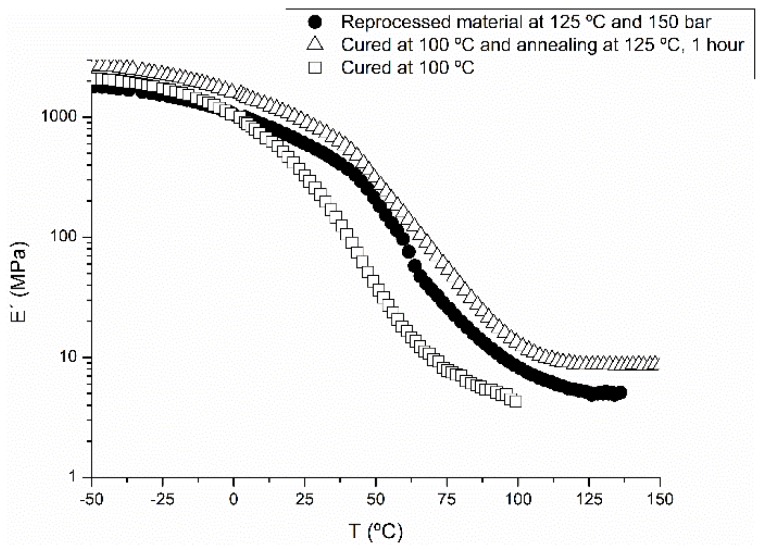
Storage modulus of the samples 5050DSS before reprocessing, after reprocessing, and after annealing at 125 °C for 1 h.

The properties of the reprocessed film were analysed by DMA experiments (Figure 14).

As observed, the storage modulus of the sample increased in the reprocessing cycle. In order to explain this behavior and taking into consideration that the reprocessing was performed at a temperature higher than the curing, the non-reprocessed sample was annealed at the reprocessing temperature and the storage modulus was measured. As observed, the modulus after the annealing was similar to that of the reprocessed sample which could suggest the degradation of the sample. In order to explain this behavior the infrared spectra of the sample before and after annealing were collected (see Supplementary Material Figure S11). As observed, the absorbance of the band at 915 cm^−1^ was reduced after annealing, which was indicative of an increase in the curing degree from 86% to 95%. Therefore, it could be concluded that the change in the storage modulus of the reprocessed sample was probably due to an increase in the crosslinking density and, therefore, the sample could be reprocessed.

Finally, the shape memory properties of the recycled material were tested visually. (Figure S12, supporting material). Surprisingly, the recycled material showed shape memory properties. This confirmed the fact that the epoxy network was restored during reprocessing. However, the fixity and recovery ratios calculated by DMA in the recycled sample (Table 2 and Figure S13) were lower than those of the pristine sample. This was related with the increase of the curing degree that happened during the reprocessing cycle, which increased the storage modulus. As described previously for the samples containing DDM, at the test temperature (80 °C), the recycled sample was between the glassy and the rubbery state and, thus, the recovery and fixity processes were less effective.

## 3. Materials and Methods 

### 3.1. Materials

Bisphenol A diglycidyl ether (DGEBA, Mw = 340.41 g/mol) and bis-(4-aminophenyl)-methane 97% (DDM) were supplied by Sigma Aldrich, Madrid, Spain. 4.4’-dithiodianiline (DSS) was supplied by Tokyo Chemical Industry, Zwijndrecht, Belgium. Linear poly(ε-caprolactone) (PCL, Mn = 50,000 g/mol) was purchased from Perstorp, Malmö, Sweden.

### 3.2. Synthesis

DGEBA and PCL were poured into a 50 mL round bottom flask. The mixtures were heated at 100 °C and mechanically stirred at 50 rpm for 1 h, to obtain a homogeneous mixture. When the solution was homogeneous the curing agents were added, and the temperature and stirring were maintained for additional 30 min. Table 3 summarizes the composition of the prepared samples.

Films of the DGEBA/PCL mixtures were made by compression molding (Specac M27544, Orpington England) with a film maker and a heating device (Specac 4000 Series High Stability Temperature Controller). A total of 0.2–0.3 g of the mixture was heated at 100 °C without pressure for 5 min, and afterwards a pressure of 5 tons was applied for 5 min, while maintaining the temperature. Using this procedure, films of a thickness of 500 μm were obtained. To perform the curing process, these films were placed in an oven (Selecta Dryterm 2000787, Barcelona, Spain) at 100 °C for 16 h.

The material was reprocessed by compression molding. The grinded material was pressed inside a mold at 150 bars at 125 °C, for 1 h.

### 3.3. Characterization

The curing process was chemically characterized by Fourier transform infrared spectroscopy (Nicolet 6700 FTIR) equipped with a single reflection Attenuated Total Reflectance ATR system (Specac Golden Gate, Orpington England). It was then followed by means of the absorbance decrease in the band centered at 915 cm^−1^, which was assigned to the out-of-plane bending vibration of the epoxy group.

The curing process was also studied by differential scanning calorimetry experiments (DSC TA Instruments Q2000, New Castle DE USA). Samples were heated from −80 °C to 300 °C, at a heating rate of 10 °C min^−1^, in the standard mode. To study the crystallization process, the samples were heated from −80 to 150 °C.

The morphology of the blends was studied by Scanning Electron Microscope (SEM) (Hitachi S-2700 with 15 kilovoltage, Chiyoda, Japan), with a fine cover of gold particles.

Dynamic mechanical properties were studied by DMA (Eplexor 100 N, Gabo Qualimeter, Selb, Germany). To obtain the storage modulus vs. temperature, 5 mm × 10 mm specimens were scanned from 0 °C to 150 °C, at a frequency of 1 Hz, a strain of 0.5% and a heating rate of 2 °C min^−1^.

### 3.4. Shape Memory Properties

The shape memory properties were studied by visual methods and by DMA. 

a) Visual shape memory

Totally cured films were placed in water at 80 °C for 10 s. Then, a deformation was applied to the material, which enabled it to programme a temporary shape. This temporary shape was fixed by introducing it in water at room temperature, for 20 s. Finally, the original shape was recovered when the sample with the temporary shape was introduced again in water at 80 °C. 

b) DMA testing

Shape memory test were performed employing a DMA Q800 (TA Instruments, New Castle, DE USA) in a force-controlled mode. Samples with dimensions of 5 × 10 mm and thickness of around 500 μm were tested. The shape memory test was performed in three steps.

(a) Step 1—Programming. The sample with an initial deformation (εi) was kept at 80 °C for 5 min. After this isothermal step, a static force was applied to achieve a strain of 10%. This was considered to be the programmed elongation (εp).

(b) Step 2—Fixation. The sample was cooled from 80 °C to −20 °C at 3 °C min^−1^, while maintaining the force as well as temperature for 5 min. The elongation obtained after relaxing the force was recorded as the fixed elongation (εf).

(c) Step 3—Recovery. The sample was heated, free of tension, up to 80 °C at a 5 °C min^−1^ rate. Once the final temperature was reached, the sample was maintained at this temperature for 30 min, and the final elongation was recorded as the recovery elongation (εr).

The fixity and recovery ratios were calculated by employing Equations (3) and (4.)

(3) $$Fixity\;\%=\frac{\varepsilon_{f}-\varepsilon_{i}}{\varepsilon_{p}-\varepsilon_{i}}\times 100$$

(4) $$Recovery\;\%=\frac{\varepsilon_{f}-\varepsilon_{r}}{\varepsilon_{f}-\varepsilon_{i}}\times 100$$

where εi, εp, εf and εr are the initial, programmed, fixed, and recovery elongation, respectively.

The first cycle was performed three times. In addition, three cycles were performed for each sample.

## 4. Conclusions

Shape memory materials were successfully prepared by using DGEBA/polycaprolactone blends cured with conventional and reversible curing agents. The shape memory properties were governed by the glass transition temperature of the epoxy that could be tailored by introducing different amounts of PCL. 

The introduction of disulfide moieties by means of the curing agent allowed for the shape memory materials, whose permanent shape could be changed when the disulfide DDM curing agent ratio obtained was at least 50/50. The sample containing only the disulfide curing agent was able to be reprocessed in a new film that presented shape memory properties.

## Figures and Tables

**Table 1:** Crystallinity of different composition DGEBA/PCL blends cured with DDM.

Sample	Melting Enthalpy (J/g) Second Scan	Crystallinity (%)
6040DDM	1	0.7
5050DDM	3.4	2.5
4060DDM	4.6	3.4
PCL	53.7	40.9

**Table 2:** Fixity and recovery ratios of different samples cured with DDM/DSS tested by DMA.

Sample	Fixity Ratio (%)			Recovery Ratio (%)		
	Cycle 1	Cycle 2	Cycle 3	Cycle 1	Cycle 2	Cycle 3
5050DDM	80	68	65	57	90	95
5050DDMDSS	92	80	79	53	96	97
5050DSS	91	88	88	85	98	98
5050DSS Reprocessed	86	80	79	62	89	91

**Table 3:** Composition of the prepared samples.

Sample	DGEBA (g)	DGEBA (mol)	PCL (g)	DSS (g)	DSS (mol)	DDM (g)	DDM (mol)
4060DDM	4	11.7	6	0	0	1.16	5.85
5050DDM	4	11.7	4	0	0	1.16	5.85
5050DDMDSS	4	11.7	4	0.72	2.92	0.58	2.92
5050DSS	4	11.7	4	1.45	5.85	0	0
6040DDM	4	11.7	2.67	0	0	1.16	5.85

## Supplementary Materials

The following are available online, Figure S1. Infrared spectra of sample 5050DDM at different curing times. Figure S1. Infrared spectra of sample 5050 DDM at different curing times. Figure S2; Scale expanded infrared spectra of sample 5050DDM at different curing times. Figure S3. Scale expanded Infrared spectra of sample 5050DDM with the integration limits. Figure S4. Conversion vs. time calculated from DSC scan for samples containing different DGEBA/PCL ratios, cured with DDM. Figure S5. Tan δ vs. temperature for DGEBA cured with DDM. Figure S6. Conversion vs. time calculated from DSC run 50/50 DGEBA/PCL blends cured with different DDM/DSS ratios. Figure S7. Tan δ vs. temperature for DGEBA cured with DSS. Figure S8. SEM images of the sample 5050DSS (a) x2.00k magnification and (b) x3.00k. Figure S9. DMA shape memory test for sample 5050DDM. Figure S10. DMA shape memory test for sample 5050DDMDSS. Figure S11. Scale expanded infrared spectra of sample 5050DSS, before and after annealing. Figure S12. Shape memory cycle for the reprocessed sample 5050 DSS. Figure S13. DMA shape memory test for the reprocessed sample 5050 DSS. Table S1. Infrared areas and conversion at different times for samples 4060DDM, 5050DDM, and 6040DDM. Table S2. Enthalpy and conversion of different composition DGEBA/PCL blends cured at different times. Table S3. Infrared absorption and conversion at different times for different samples. Table S4. Enthalpy and conversion at different times for different samples.
