# Supplementary material for: Reprogrammable Permanent Shape Memory Materials Based on Reversibly Crosslinked Epoxy/PCL Blends
Source: Molecules. 2020 Mar 29;25(7):1568. doi: 10.3390/molecules25071568 (PMC7180467; doi:10.3390/molecules25071568)
Supplement: Supplementary file 1 [file molecules-25-01568-s001.pdf]

Supplementary Material:

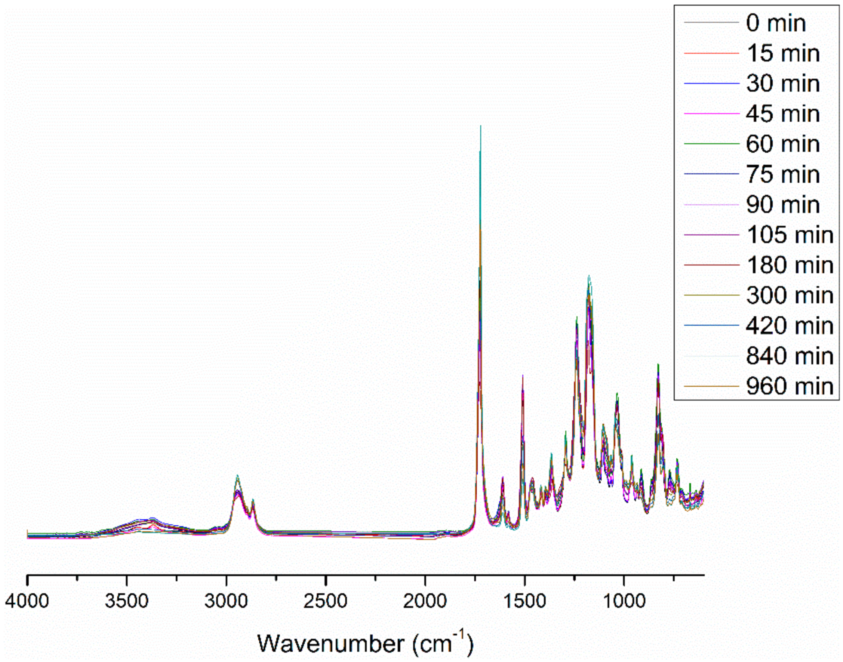

Figure S1. Infrared spectra of sample 5050DDM at different curing times.

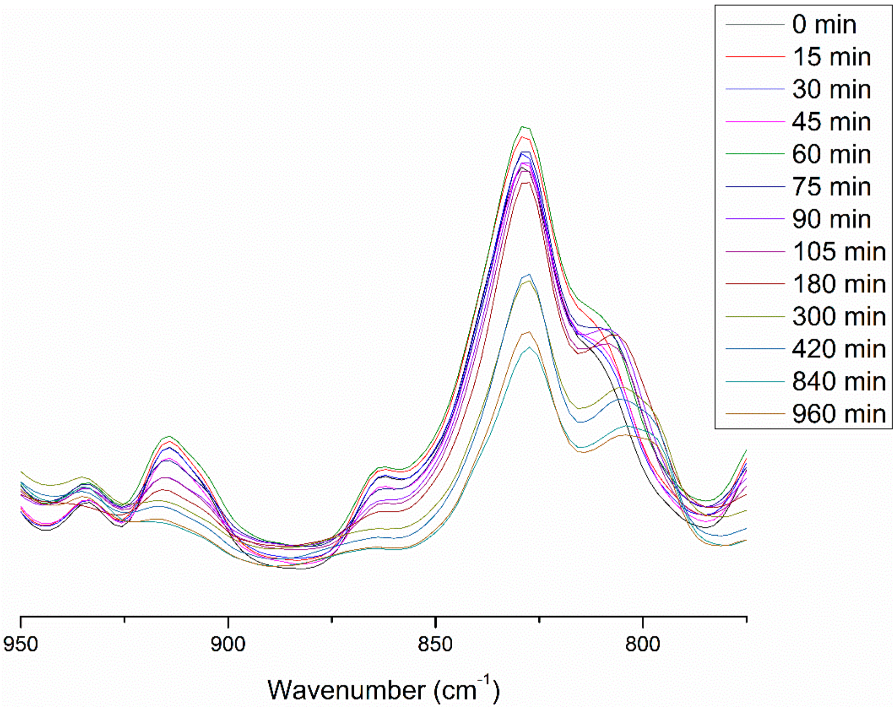

Figure S2. Scale expanded Infrared spectra of sample 5050DDM at different curing times.

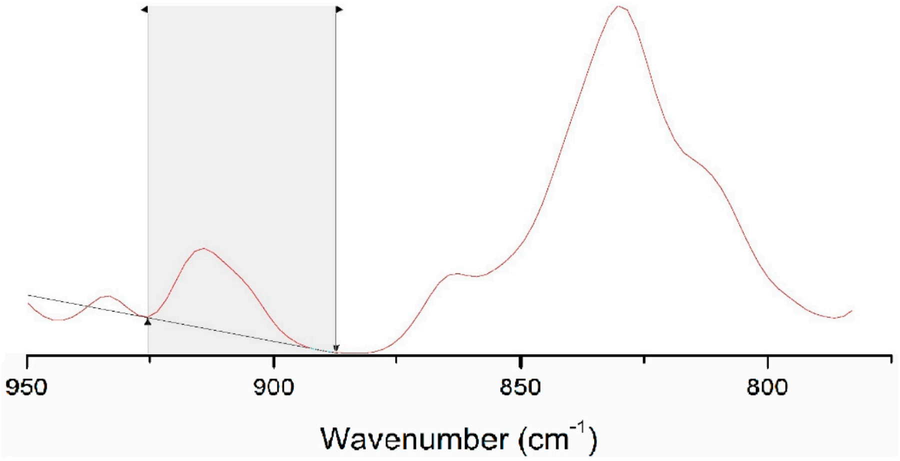

Figure S3. Scale expanded Infrared spectra of sample 5050DDM with the integration limits.

Table S1. Infrared areas and conversion at different times for samples 4060DDM, 5050DDM and 6040DDM.

	4060DDM			5050DDM			6040DDM		
t (h)	A 915 cm^{-1}	A 830 cm^{-1}	X	A915 cm^{-1}	A830 cm^{-1}	X	A915 cm^{-1}	A830 cm^{-1}	X
0	0.879	6.125	0	1.11	7.12	0	1.077	9.019	0
0.25	0.853	6.307	6	1.09	7.74	9	0.803	9.805	31
0.5	0.784	6.233	12	1.01	7.13	8	0.686	9.655	41
1	0.746	6.112	15	0.92	7.43	20	0.435	9.968	60
1.25	0.510	6.487	45	0.98	7.85	19	0.467	8.418	53
1.5	0.407	6.608	57	0.68	7.67	42	0.391	8.957	63
1.75	0.750	6.408	-	0.47	7.68	61	0.290	8.815	72
3.5	0.414	6.007	52	0.49	7.47	58	0.191	9.135	83
5	0.329	5.961	62	0.36	7.41	69	0.114	8.276	88
7	5.77E-02	4.239	91	0.07	5.74	86	0.671	5.061	89
14	0	2.868	-	0.13	6.06	99	0.128	8.156	87
16	5.47E-02	4.181	91	0.00	4.97	92	0.047	4.021	93

Table S2. Enthalpy and conversion of different composition DGEBA/PCL blends cured at different times

	**40/60DDM**		**5050DDM**		**6040DDM**	
**t (h)**	Enthalpy (J/g)	Conversion	Enthalpy (J/g)	Conversion	Enthalpy (J/g)	Conversion
**0**	180	0	239	0	235	0
**0.5**	110	39	168	29	142	39
**1.5**	84	53	151	37	64	72

**1.75**	83	54	131	45	59	74
**7**	73	59	34	86	21	91
**16**	28	84	21	91	21	91

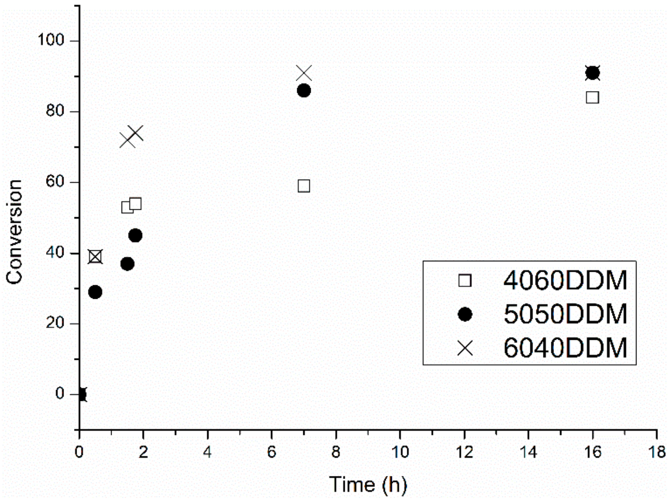

Figure S4. Conversion vs time calculated from DSC scan for samples containing different DGEBA/PCL ratios cured with DDM.

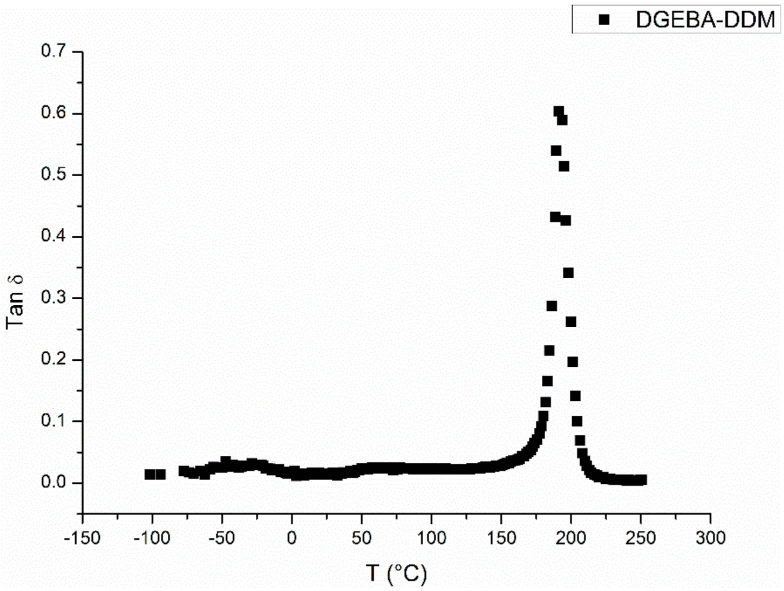

Figure S5. Tan δ vs temperature for DGEBA cured with DDM.

Table S3. Infrared absorption and conversion at different times for different samples

	5050DSS			5050DDMDSS		
t (h)	A 915 cm^{-1}	A 830 cm^{-1}	X	A 915 cm^{-1}	A 830 cm^{-1}	X
0	1.273	8.923	0.00	1.260	7.773	0
0.25	1.192	8.351	0.00	1.179	7.599	0
0.5	1.135	8.393	6	1.190	7.759	1

0.75	1.126	8.462	7	1.165	7.783	4
1	1.104	8.499	9	1.061	7.95	14
1.25	1.115	8.567	9	1.111	8.353	14
1.5	1.122	8.757	10	1.014	8.095	19
1.75	0.974	8.838	23	0.659	8.327	49
3.5	0.629	9.584	55	0.539	8.360	58
5	0.795	9.446	41	0.335	8.220	74
7	0.262	8.945	80	0.389	8.537	71
14	-0.051	4.454	107	5.50E-02	5.97	94
16	0.171	8.812	86	0	4.914	1

Table S4. Enthalpy and conversion at different times for different samples.

	**5050DDM**		**5050DDMDSS**		**5050DSS**	
**t (h)**	Enthalpy (J/g)	Conversion	Enthalpy (J/g)	Conversion	Enthalpy (J/g)	Conversion
**0**	239	0	225	0.00	216	0
**0.5**	168	29	168	25	242	0
**1.5**	151	37	157	30	236	0
**1.75**	131	45	15	33	173	20
**7**	34	86	37	83	73	66
**16**	21	91	28	87	42	80

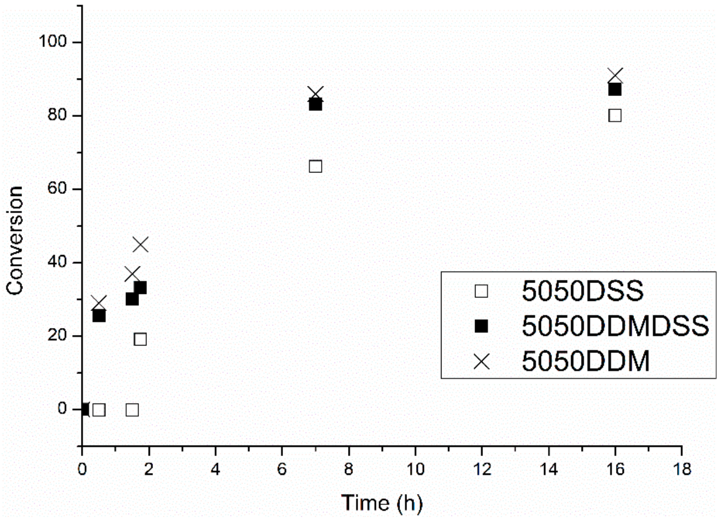

Figure S6. Conversion vs time calculated from DSC run 50/50 DGEBA/PCL blends cured with different DDM/DSS ratios.

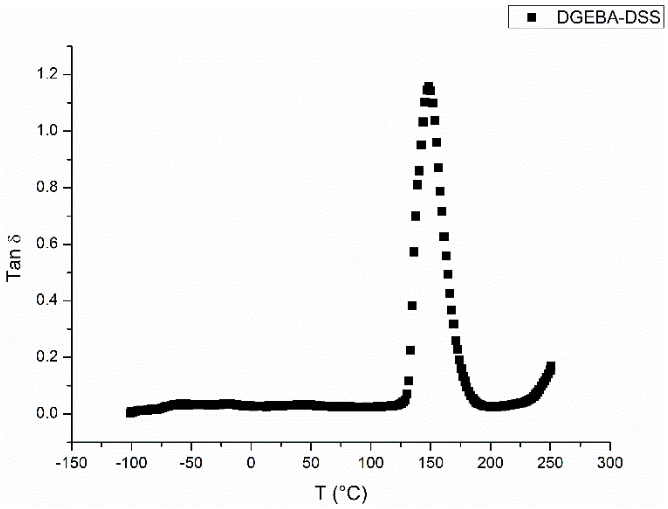

Figure S7. Tan δ vs temperature for DGEBA cured with DSS.

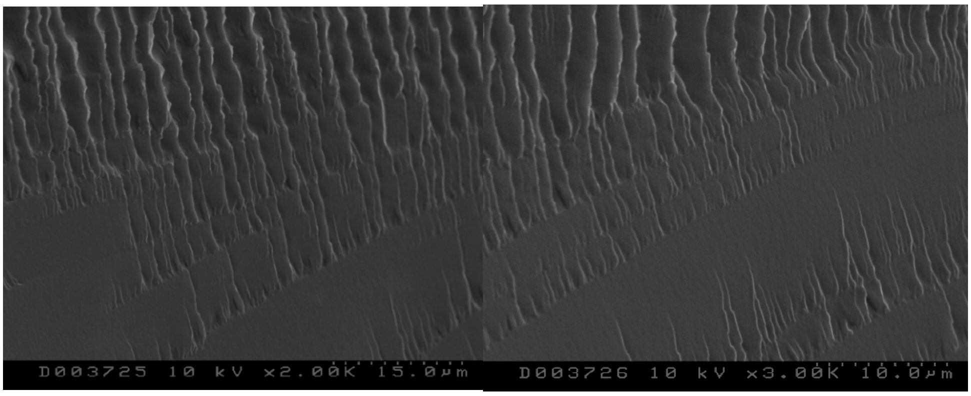

(a) **(b)**

Figure S8. SEM images of the sample 5050DSS. (a) x2.00k magnification and b) x3.00k magnification.

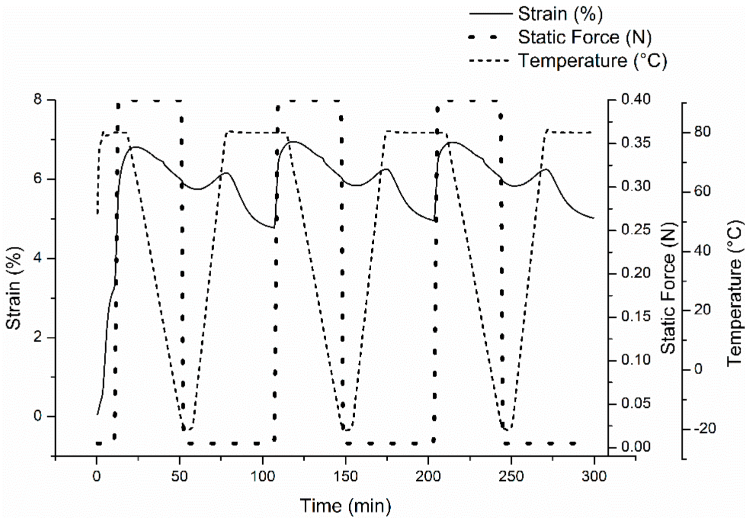

Figure S9. DMA shape memory test for sample 5050DDM

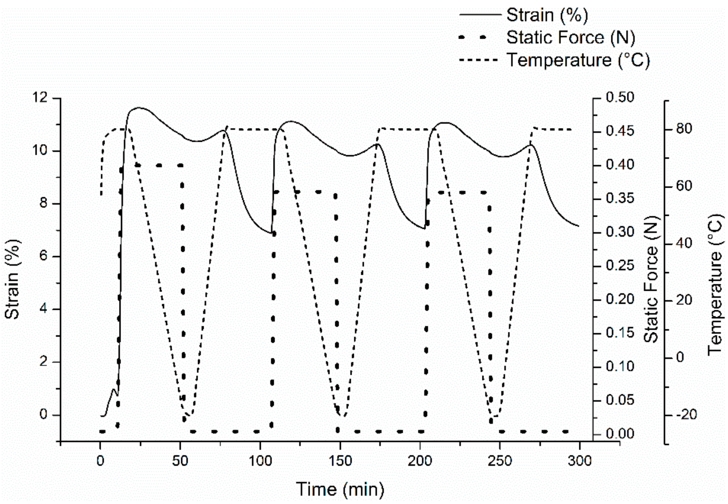

Figure S10. DMA shape memory test for sample 5050DDMDSS

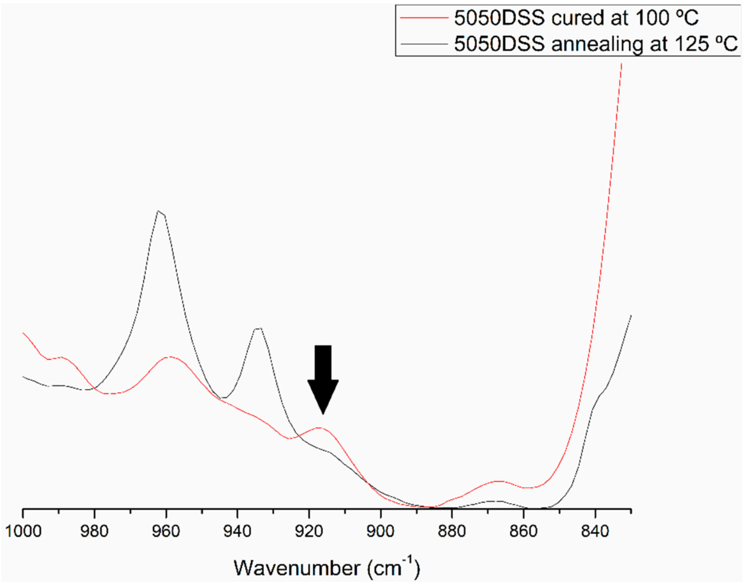

Figure S11. Scale expanded Infrared spectra of sample 5050DSS before and after annealing.

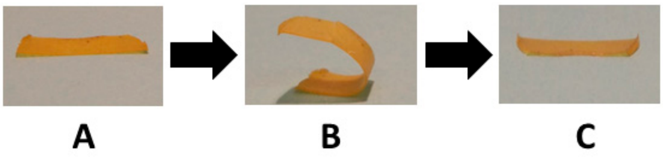

Figure S12. Shape memory cycle for the reprocessed sample 5050 DSS.

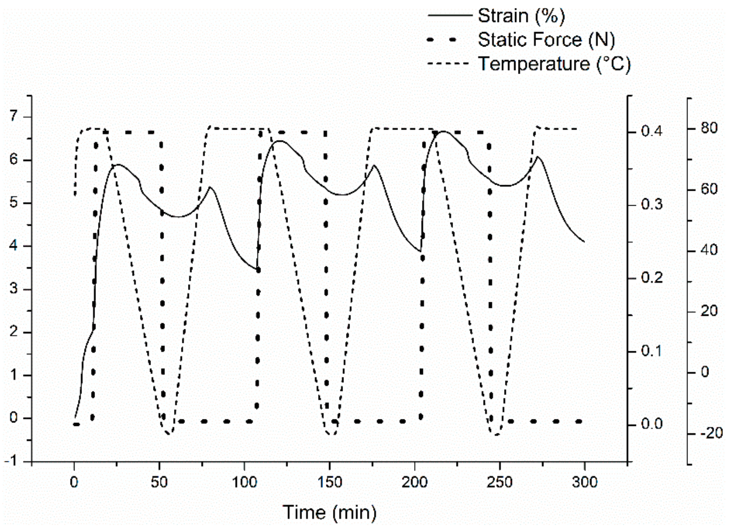

Figure S13. DMA shape memory test for the reprocessed sample 5050 DSS.
